# The functional gait deviation index

**DOI:** 10.1080/02664763.2025.2514150

**Published:** 2025-06-11

**Authors:** Sajal Kaur Minhas, Morgan Sangeux, Julia Polak, Michelle Carey

**Affiliations:** a School of Mathematics and Statistics, University College Dublin, Belfield, Ireland; b University Children’s Hospital Basel, Department of Orthopaedics, Basel, Switzerland; c School of Mathematics and Statistics, University of Melbourne, Melbourne, Australia

**Keywords:** Kinematics, multivariate functional principal components, gait pathology, functional data analysis, biomechanics, 62R10, 62H25

## Abstract

A typical gait analysis requires the examination of the motion of nine joint angles on the left-hand side and six joint angles on the right-hand side across multiple subjects. Due to the quantity and complexity of the data, it is useful to calculate the amount by which a subject’s gait deviates from an average normal profile and to represent this deviation as a single number. Such a measure can quantify the overall severity of a condition affecting walking, monitor progress, or evaluate the outcome of an intervention prescribed to improve the gait pattern. The gait deviation index, gait profile score, and the overall abnormality measure are standard benchmarks for quantifying gait abnormality. However, these indices do not account for the intrinsic smoothness of the gait movement at each joint/plane and the potential co-variation between the joints/planes. Utilizing a multivariate functional principal component analysis we propose the functional gait deviation index (FGDI). FGDI accounts for the intrinsic smoothness of the gait movement at each joint/plane and the potential co-variation between the joints. We show that FGDI scales with overall gait function, provides a consistent measure of gait abnormality, and is implemented easily using an interactive web app.

## 1. Introduction

Typical gait data capture the movement of key kinematic variables throughout an individual’s stride. These variables encompass pelvic and hip angles across all three planes, knee flexion/extension, ankle dorsiflexion/plantarflexion, and foot internal/external rotation. Measurements are taken at 1% intervals throughout the entire 100% gait cycle. A prevalent objective in this analysis is to determine the extent to which an individual’s gait pattern deviates from the average gait pattern observed in a group of healthy subjects.

**Figure 1.**
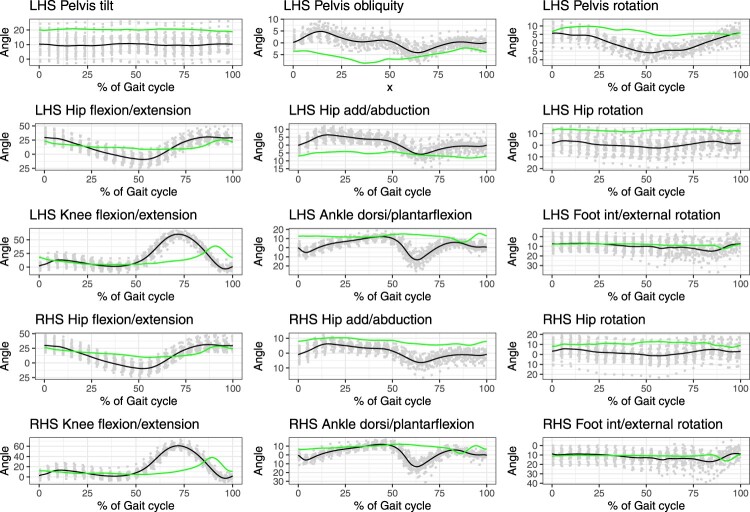
The gait patterns of 42 healthy subjects (represented in grey) and their mean (shown in black), alongside the gait pattern of a subject with Parkinson’s disease (highlighted in green).

Figure 1 presents an illustrative example, displaying the gait patterns of 42 healthy subjects (depicted in grey) and their mean (shown in black), alongside the gait pattern of a subject with Parkinson’s disease (highlighted in green). This figure includes nine joint angles on the left-hand side and six on the right-hand side^1^. It is important to note that since the pelvis is common to both sides, it is appropriate to include pelvic kinematics from only one side.

As illustrated in Figure 1, the gait data are highly interdependent, with many kinematic variables exhibiting similar behavior across the gait cycle, planes, and joints. However, subjects with conditions affecting their walking patterns show significant differences, primarily due to variations in the timing and amplitude of their gait waveforms. Given the substantial amount and complexity of the data, there is a preference for employing a single metric to assess the extent of deviation in an individual’s walking pattern from the average pattern observed in a control group. This metric can be useful for assessing the overall severity of a walking impairment, tracking improvements over time, or evaluating the effectiveness of interventions aimed at enhancing walking patterns.

The Gait Deviation Index (GDI) and the Gait Profile Score (GPS) are established indices for assessing gait abnormalities, as detailed in Schwartz (2008) and Baker (2009), respectively. The GDI employs singular value decomposition on kinematic data from a reference database that includes all children who received gait analysis at the Gillette Children’s Specialty Healthcare Center between February 1994 and April 2007. This decomposition generates fifteen independent gait features that form the basis for projecting the kinematic variables of the subjects within any independently collected gait data sample. The GDI is calculated by measuring the distance between a subject’s projected kinematics and the average projected kinematics of the control group. GDI provides a metric for ranking individuals based on their deviation from the average behavior of the control group, offering distinct severity measures for the left and right legs.

The GPS is derived directly from the collected gait data sample as the root mean square difference between a subjects’s kinematic data and the average kinematic data of a control group. This measure provides an evaluation of gait abnormalities for both legs combined, each leg separately, and at the individual joint level. Such a detailed breakdown enhances understanding of which specific legs or joints contribute to gait abnormalities, thereby facilitating more targeted and effective treatment planning.

Although the GDI and GPS have been effective in assessing common gait pathologies such as cerebral palsy [17,21], rheumatoid arthritis [5], and Parkinson’s disease [8], they present inherent challenges. Kinematic variables for any individual are often highly correlated; the motion of one joint can significantly influence the motion of adjacent joints. Furthermore, the position of a joint at one point in the gait cycle strongly influences its position in later instances. This interdependence of kinematic data may result in biased assessments of overall abnormality when using the GDI and GPS indices, as demonstrated in [16].

The Overall Abnormality (OA) approach, introduced by Marks *et al.* [16], addresses the limitations inherent in the GDI and GPS. In contrast to the GDI, which derives its gait features from a historical reference database, the OA method applies principal component analysis (PCA) to kinematic data collected for the study’s control group, thereby deriving *K* independent gait features. A subject’s kinematic variables are then projected into this feature space, and the OA metric calculates the distance between the subject’s projected kinematics and the average of the projected kinematics for the control group. This method assesses severity across both legs, individually for each leg, and at each joint. However, utilizing PCA solely on the control group may lead to biased abnormality estimates, as the control group’s gait features might not adequately represent the patterns observed in subjects with significantly abnormal gaits. Moreover, while PCA recognizes the high interdependence of kinematic data, it does not fully address the essential structure of this dependence. The temporal sequence of kinematic data throughout the gait cycle is crucial in gait analysis and should be preserved. The interconnectedness across joints and planes often leads to multicollinearity, which can introduce bias in the measurement of overall abnormality.

In gait analysis, measurements are denoted by $m_{i,j,l}$, where *j* ranges from 1 to 18, covering the pelvis and hip angles across all three planes, knee flexion/extension, ankle dorsiflexion/plantarflexion, and foot internal/external rotation for both the left and right sides. The index *i* represents individual subjects, numbered from 1 to *N*, and *l* indicates the observed points throughout the gait cycle, from 1 to *T*. To encapsulate the temporal ordering and the continuous nature of movement, a commonly employed approach [4,12,23,25] treats the set of discrete measurements $m_{i,j,l}$ as noisy realizations of a unified entity, a curve $m_{i,j}(t)$, representing the movement across the entire gait cycle *t* for each individual *i* and kinematic variable *j*. By aggregating these curves, we obtain a multivariate functional data set, so that for each individual a set of 18 functions is observed representing the movement for each of the 18 kinematic variables.

Multivariate Functional Principal Components Analysis (MFPCA) identifies the principal modes of variation within the covariance structure of the multivariate functional data set, accounting for potential covariations among the multiple kinematic variables. These modes represented as eigenfunctions of the covariance operator, capture significant deviations from the mean behavior of each kinematic variable. The principal component scores derived from these eigenfunctions provide insights into individual deviations from typical behavior. MFPCA effectively addresses potential multicollinearity issues due to interdependencies between joints or planes while preserving the temporal ordering throughout the gait cycle.

Applying MFPCA to the functional dataset yields independent gait features, into which any subject’s kinematic variables can be projected. Similar to the GDI and OA approaches, the proposed FGDI is calculated by measuring the distance between a subject’s projected kinematics and the average of the projected kinematics of the control group. Recently, Roach *et al.* [24] and Yoshida *et al.* [29] performed MFPCA on kinematic data and examined the resulting functional principal component scores to gain insight into the differences between two cohorts of individuals. These approaches differ from the proposed approach as they do not provide a single measure that can be used to quantify the overall severity of a condition or a means to rank the subjects based on gait pathology.

FGDI combines the advantages of the existing GDI, GPS and OA approaches. As the FGDI index is easy to interpret and can provide a measure of severity on both legs, as well as on each leg and at each joint separately. Additionally, the proposed approach accounts for the structure of the dependence of human gait leading to a more accurate quantification of gait pathology which can improve clinical decision-making.

## 2. Quantifying gait abnormalities

As discussed in the introduction, the conventional metrics used to quantify gait abnormalities include the GDI [26], the GPS [2], and the OA approach [16]. For more details on these approaches, please consult Sections A.1, A.2, and A.3 in Appendix 1.

In this section, we will begin by demonstrating how to apply the MFPCA technique, as described in [11], within the context of gait data. MFPCA is employed to identify the dominant modes of variation among individuals functions representing gait at each joint and within each plane. Subsequently, we will discuss the utilization of results from the MFPCA to compute the FGDI.

In gait analysis, data collection typically involves the recording of all 18 kinematic variables. However, subsequent analyzes focus only on specific subsets of these variables, tailored to the particular objectives of the study. For the computation of the FGDI, we concentrate on three distinct subsets of these variables, which are outlined as follows:

1. Combined Approach: The first method involves using the combined observations from nine kinematic variables for the left side and six kinematic variables for the right side. Given that the pelvis is common to both sides, we include pelvic kinematics from one side only. This procedure results in the selection of fifteen kinematic variables, designated as u=1,…,15. This approach yields a measure of severity by collectively considering both legs, thereby providing an overall assessment of gait abnormality.
2. Individual Leg Approach: The FGDI can be calculated separately for each leg by utilizing the observations from the nine kinematic variables specific to that leg, designated with indices u=1,…,9. This method provides a measure of gait pathology for each leg individually, facilitating a detailed assessment of gait abnormality in each leg.
3. Joint/Plane Specific Approach: The FGDI can be computed for each kinematic variable separately, resulting in a measure of severity for each joint or plane, denoted by u=1. This method offers a detailed evaluation of gait abnormalities at the individual level of each joint or plane.

Let the specific subset of kinematic variables be denoted by u=1,…,U, where *U* represents either 15, 9, or 1, as described above.

### 2.1. Multivariate functional principal component analysis

For each subject *i*, and kinematic variable *u*, the movement throughout the entire gait cycle can be expressed as follows:

(1) $$m_{i,u,l}=m_{i,u}(t_{l})+\epsilon_{i,u,l},\;\mathrm{for}\;i=1,\ldots ,N,\;u=1,\ldots ,U,\;\mathrm{and}\;l=1,\ldots ,T.$$

where $m_{i,u}(t_{l})$ denotes the curve describing the movement of the *u*th kinematic variable for subject *i* evaluated at point $t_{l}$ within the gait cycle. The term $\epsilon_{i,u,l}$ represents the measurement error, which is presumed to follow a normal distribution with a mean of zero and constant variance $\sigma^{2}$. The function $m_{i,u}(t)$ represents the inherent smoothness of gait movement throughout the entire gait cycle *t*. Let ${\tilde{m}}_{i,u}(t)=m_{i,u}(t)-\mu_{u}$ represent the centered gait data for the *i*th subject and the *u*th kinematic variable, observed at the *T* points over the gait cycle t. Here, $\mu_{u}$ denotes the mean of $m_{i,u}(t)$ across all subjects for the *u*th kinematic variable. The algorithm for obtaining the multivariate functional principal components, as outlined in [11], then proceeds with the following two steps:

1. Calculate a univariate functional PCA on ${\tilde{m}}_{i,u}(t)$ for each kinematic variable *u*. Here we use the functional PCA with fast covariance estimation attributable to [28]. This results in principal component functions ${\hat{\phi }}_{1}^{u},\ldots ,{\hat{\phi }}_{K_{u}}^{u},$ and principal component scores, ${\hat{\xi }}_{i,1}^{u},\ldots ,{\hat{\xi }}_{i,K_{u}}^{u},$ for each subject i=1,…,N and each kinematic variable u=1,…,U. The truncation lag for each variable, $K_{u},$ is chosen as the number of components that have a proportion of variance explained greater than *ω*, where *ω* is typically 0.99. When implementing the Joint/Plane Specific Approach, proceeding to step two is not necessary, as this method involves considering only a single joint or plane.
2. Find the dominant modes of variation across multiple joints/planes: Combine all the principal component scores from each joint/plane into one big matrix $\Xi \in R^{N\times K_{+}}$ with $K_{+}=\sum_{u=1}^{U}K_{u}$ having rows $\Xi_{i}=({\hat{\xi }}_{i,1}^{\left(1\right)},\ldots ,{\hat{\xi }}_{i,K_{1}}^{\left(1\right)},\ldots ,{\hat{\xi }}_{i,1}^{\left(U\right)},\ldots ,{\hat{\xi }}_{i,K_{U}}^{\left(U\right)}),$ and estimate the joint covariance matrix $\hat{Z}=\frac{1}{N-1}\Xi^{T}\Xi .$ Perform a matrix eigen-analysis on $\hat{Z},$ to determine the eigenvectors ${\hat{\kappa }}_{w}$ and eigenvalues ${\hat{\nu }}_{w}$ of $\hat{Z}$ for w=1,…,W, for some truncation lag $W<K_{+}$. Here the truncation lag *W* is selected by the number of components that have a proportion of variance explained greater than *ω*, where *ω* is typically 0.99. The estimated multivariate orthonormal principal component scores ${\hat{\rho }}_{i,w}$ are then given by:
$${\hat{\rho }}_{i,w}=((N-1){\hat{\nu }}_{w}{)}^{1/2}({\hat{\kappa }}_{w}^{T}\Xi^{T}\Xi {\hat{\kappa }}_{w}{)}^{-1/2}\Xi_{i}{\hat{\kappa }}_{w},$$
  for w=1,…,W. The MFPCA takes the co-variation between the different joints/planes into account by weighting the univariate functional principal component scores ${\hat{\xi }}_{i,k}^{\left(u\right)}$ by the eigenvector ${\hat{\kappa }}_{w}$ of the covariance of the scores, representing the dependence across joints/planes, see [11] for further details.

### 2.2. The functional gait deviation index

Denote the N×P matrix of estimated principal component scores by $\hat{\varphi }$. If we are interested in calculating an index representing the gait abnormality at each joint/plane (the joint/plane specific approach) then we use the univariate principal component scores in Step 1 in Section 2.1. That is ${\hat{\varphi }}_{i,k}={\hat{\xi }}_{i,k}^{\left(u\right)},$ for $k=1,\ldots ,K_{u},$ which implies $P=K_{u}$. If we are interested in calculating an index representing the gait abnormality across multiple joints/planes accounting for the co-variation between the joints/planes (i.e. the individual leg or combined approach) then we use the multivariate principal component scores in Step 2 of the algorithm provided in Section 2.1. That is ${\hat{\varphi }}_{i,w}={\hat{\rho }}_{i,w}$ for w=1,…,W, which implies *P* = *W*.

First, calculate the average of the principal component scores from all healthy subjects, ${\bar{\hat{\varphi }}}_{p}^{\left(H\right)},$ for p=1,…,P. Then calculate the squared distance from the $p^{\mathrm{th}}$ principal component score for the $i^{\mathrm{th}}$ subject, ${\hat{\varphi }}_{i,p}$, to the average $p^{\mathrm{th}}$ principal component scores of all healthy subjects, which we denote by $d_{i,p}=({\hat{\varphi }}_{i,p}-{\bar{\hat{\varphi }}}_{p}^{\left(H\right)}{)}^{2}.$ For details concerning the distribution of $d_{i,p}$, please refer to Appendix 2. The FGDI for subject *i* is then given by the logarithm of the square root of the sum of the distances

$${\mathrm{FGDI}}_{i}=\log\left(\sqrt{\sum_{p=1}^{P}d_{i,p}}\right).$$

The FGDI can be used in its raw format as a measure of gait pathology. Akin to GDI, to improve interpretation, we can scale the ${\mathrm{FGDI}}_{i}$. Compute the sample mean and standard deviation of the FGDI for the healthy subjects denoted by $\mu_{{\mathrm{FGDI}}_{H}}$ and $\sigma_{{\mathrm{FGDI}}_{H}}$ respectively. Then determine the z-score with respect to the healthy subjects

(2) $${\mathrm{sFGDI}}_{i}=\frac{{\mathrm{FGDI}}_{i}-\mu_{{\mathrm{FGDI}}_{H}}}{\sigma_{{\mathrm{FGDI}}_{H}}}.$$

The scaled FGDI, ${\mathrm{sFGDI}}_{i},$ in (2) can be interpreted as:

- Higher values of ${\mathrm{sFGDI}}_{i}$ signify greater gait pathology.
- An ${\mathrm{sFGDI}}_{i}$ value around 0 suggests a gait similar to that of an average healthy individual, indicating minimal or no gait pathology.
- Each unit deviation from 0 in the ${\mathrm{sFGDI}}_{i}$ score represents an additional standard deviation from the average FGDI of healthy subjects. A positive deviation implies that the subject’s gait abnormality surpasses the typical level found in healthy individuals. Conversely, a negative deviation suggests that the subject’s gait does not exhibit abnormalities exceeding those typically observed in healthy individuals.

## 3. Assessing the severity of gait impairment in Parkinson’s disease patients

Parkinson’s disease (PD) is a widely prevalent, progressive neurodegenerative disorder characterized by substantial motor control impairments, including bradykinesia, tremors, rigidity, and gait instability. Global estimates in 2019 indicated that over 8.5 million individuals are affected by PD, representing a doubling of prevalence over the past 25 years [22]. Gait analysis is routinely utilized in clinical practice for severity stratification, treatment evaluation, and management of the condition.

The PD dataset is publicly accessible, as detailed in [27]. It includes data from 21 right-handed idiopathic PD individuals (5 females and 16 males), characterized by the following demographics: average age of 65 ± 10 years, average height of 166.5 ± 7.1 cm, and average mass of 71.89 ± 12.37 kg. Additionally, a healthy control dataset is available from [6], comprising 42 adults (18 females and 24 males). All participants were free from orthopedic or neurological diseases that could affect their gait. Their demographics are an average age of 43 ± 19 years, height of 167.1 ± 11 cm, and mass of 67.7 ± 11.2 kg.

The Hoehn and Yahr scale [13] is an established method for assessing the progression of PD, integrating medical evaluations with reports from patients and caregivers. The scale comprises four stages: Scale 1 is characterized by unilateral symptoms with minimal impact; Scale 2 involves bilateral symptoms without balance impairments; Scale 3 denotes moderate bilateral symptoms with some postural instability, though independence is retained; Scale 4 indicates severe disability, yet the ability to walk or stand is preserved. In the PD dataset, the distribution includes one subject at Scale 1, twelve at Scale 2, seven at Scale 3, and one at Scale 4.

The Movement Disorder Society’s Unified Parkinson’s Disease Rating Scale (MDS-UPDRS) [9] offers an alternative assessment for Parkinson’s Disease. This scale includes Part II, which evaluates motor experiences during daily activities, and Part III, which assesses motor functions including rigidity and agility. Higher scores on this scale reflect greater impairment. In the PD dataset, MDS-UPDRS Part II ranges from 1 to 13, and Part III ranges from 10 to 61.

Freezing of gait represents a notable motor symptom in PD, characterized by brief, episodic interruptions or significant reductions in the forward progression of the feet despite the intention to walk. This symptom can lead to falls and diminished independence. The dataset comprises ten patients experiencing freezing of gait (referred to as freezers) and eleven who do not exhibit this symptom (referred to as non-freezers).

For each subject, measurements of nine kinematic variables were recorded on both the left and right sides. These variables include pelvic tilt, pelvic obliquity, pelvic rotation, hip flexion/extension, hip abduction/adduction, hip rotation, knee flexion/extension, ankle dorsiflexion/plantarflexion, and foot internal/external rotation. The measurements consist of 101 equally-spaced time points throughout a single gait cycle, allowing the motion of each kinematic variable to be described in 1% increments of the gait cycle.

### 3.1. FGDI: the joint/plane specific approach

**Figure 2.**
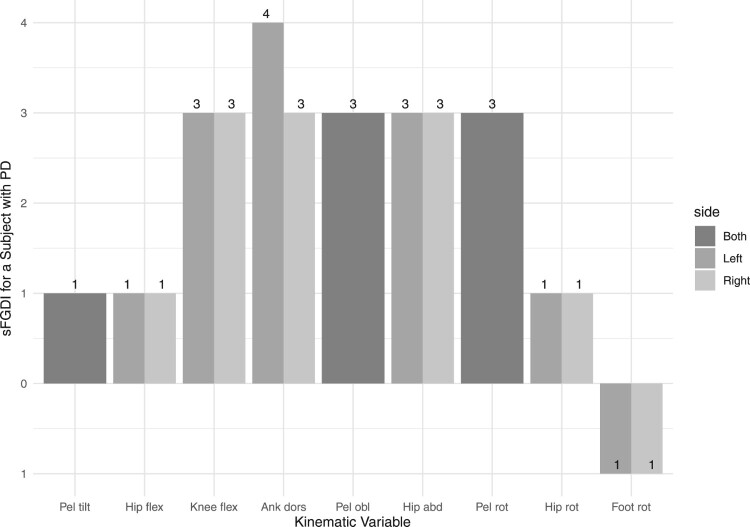
The MAP for a subject with Parkinson’s disease.

The FGDI index is calculated using univariate principal component scores as described in Subsection 2.2 with the percentage of variation explained by the univariate principal components set to 99%. This index quantifies the relative abnormality of a subject’s gait across each kinematic variable. The Movement Analysis Profile (MAP) displays the ${\mathrm{sFGDI}}_{i,u}$ in (2) for the *i*th subject and *u*th kinematic variable. The height of each bar in the MAP represents the number of standard deviations by which the subject’s ${\mathrm{FGDI}}_{i,u}$ deviates from the average FGDI of healthy subjects. For illustrative purposes, Figure 2 depicts the MAP for a subject with PD, corresponding to the kinematic data presented in Figure 1.

Figure 2 illustrates that the subject’s left-hand side ankle dorsiflexion/plantarflexion exhibits a significant deviation, measuring 4 standard deviations from the average FGDI of individuals without gait abnormalities. Conversely, the subject’s foot internal/external rotation aligns closely with standard gait patterns, as indicated by an FGDI value that is one standard deviation below the average for individuals without gait abnormalities.

It is well-established that unilateral motor symptoms are characteristic of PD, as underscored by Miller-Patterson *et al.* [20], Djaldetti *et al.* [3]. Correspondingly, most joints display comparable FGDI scores on both the left-hand side and right-hand side, except for notable discrepancies in ankle dorsiflexion/plantarflexion. This asymmetry in ankle gait patterns is consistent with recent research findings presented in [1].

Furthermore, the kinematic data presented in Figure 1 corroborates the values of the MAP, with higher values indicating trajectories, highlighted in green, that exhibit greater discrepancies from the mean of healthy individuals, shown in black. Conversely, lower values correspond to smaller discrepancies.

Table A1 in Appendix 4 demonstrates the optimal number of principal components required to account for 99% of the variance in the kinematic data, as well as 100 times the average difference in the FGDI across individuals when varying the number of principal components by plus or minus two. This analysis reveals that the FGDI remains highly stable, exhibiting minimal variations in FGDI values relative to the number of principal components.

### 3.2. FGDI: the combined leg approach

We conducted an MFPCA on both legs, extracting 50 multivariate functional principal components. These components account for 99% of the variance in the kinematic data. Following this, we calculated the scaled FGDI in (2) using the MFPCA scores as described in Subsection 2.2.

**Figure 3.**
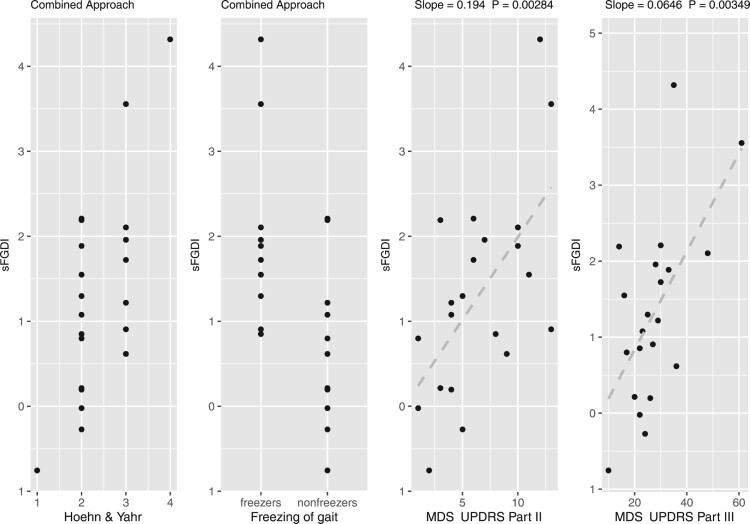
The scaled FGDI values for all PD subjects in the sample, grouped according to their Hoehn and Yahr scale, their status as either freezers or non-freezers and their scores on the MDS-UPDRS Parts II and III.

Figure 3 presents the scaled FGDI values for all PD subjects in the sample. These values are categorized according to the subjects’ Hoehn and Yahr scale classifications, their designation as either freezers or non-freezers and their scores on the MDS-UPDRS Parts II and III.

The subject exhibiting the highest scaled FGDI corresponds to the highest level on the Hoehn and Yahr scale, while the subject with the lowest scaled FGDI matches the scale’s lowest level. A Kruskal-Wallis rank sum test was conducted to evaluate whether median differences in scaled FGDI values are statistically significant relative to the Hoehn and Yahr scale, resulting in a p-value of 0.08. Subjects identified as freezers demonstrate higher scaled FGDI values compared to non-freezers, as confirmed by a Wilcoxon rank sum test with a continuity correction, which yielded a p-value of 0.007. Additionally, scaled FGDI values exhibit a linear increase with respect to the scores on Parts II and III of MDS-UPDRS, with slopes of 0.19 and 0.06 and corresponding p-values of 0.002 and 0.003, respectively.

**Figure A1.**
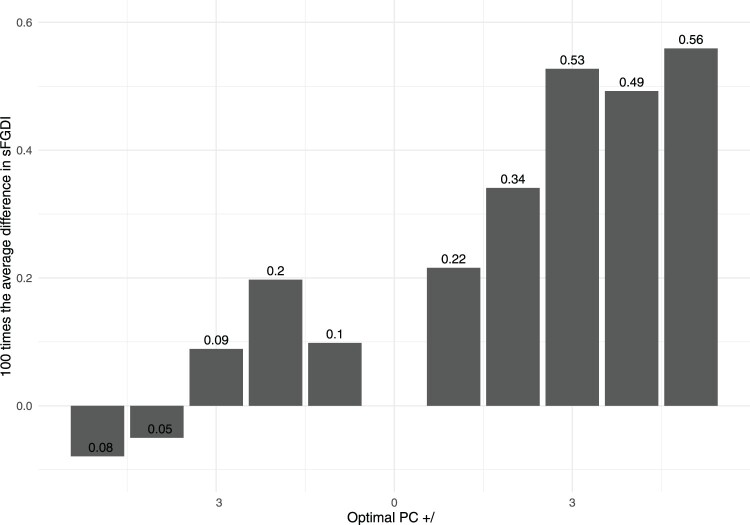
100 times the difference between the FGDI calculated with the optimal number of principal components and the FGDI obtained when the number of principal components is adjusted by ±5.

Figure A1 in Appendix 4 demonstrates 100 times the average difference in the FGDI across individuals when varying the optimal number of principal components by five. This indicates that the FGDI remains highly stable across different numbers of principal components, with minimal differences in the average values.

## 4. Assessing the severity of gait impairment in individuals with lower-limb amputations

In 2017, an estimated 57.7 million individuals worldwide were living with limb amputations resulting from traumatic causes [18]. The lower-limb amputation data are publicly accessible and can be found in [14]. This dataset includes 18 individuals with unilateral above-knee amputations, comprising 3 females and 15 males, with the following demographics: average age of 52 ± 16 years, height of 175.8 ± 9 cm, and mass of 88.85 ± 22.61 kg. A healthy control dataset, consisting of 18 females and 24 males, is publicly available from [6] and detailed in Section 2.

The Medicare Functional Classification Level, also known as K-levels, is a scale employed by private insurers to evaluate rehabilitation potential in individuals with lower limb loss. This scale ranges from 0 (lowest mobility potential when using a prosthesis) to 4 (highest mobility potential when using a prosthesis) and categorizes functional mobility and rehabilitation prospects [7,19]. Within the study cohort, nine subjects were classified as full community ambulators (K3), and nine as limited community ambulators (K2).

For each subject, measurements were recorded on both the left and right sides for nine kinematic variables: pelvic tilt, pelvic obliquity, pelvic rotation, hip flexion/extension, hip abduction/adduction, hip rotation, knee flexion/extension, ankle dorsiflexion/plantarflexion, and foot internal/external rotation. These measurements comprised 101 equally-spaced time points throughout a single gait cycle, enabling the motion of each kinematic variable to be detailed in 1% increments of the gait cycle.

### 4.1. FGDI: individual leg approach

We conducted a separate MFPCA for each leg individually (left and right). This analysis yielded 24 multivariate functional principal components for the left leg and 22 multivariate functional principal components for the right leg. These components account for 99% of the observed variability in the respective kinematic data. Subsequently, we calculated the scaled FGDI for both the left and right legs, utilizing their respective multivariate functional principal component scores as described in Section 2.2.

**Figure 4.**
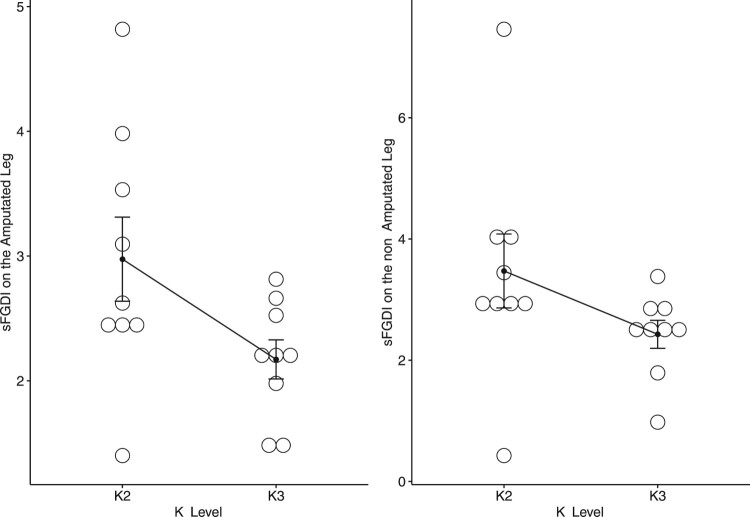
The scaled FGDI on the Amputated Leg and non Amputated Leg with respect to the K-Level.

Figure 4 displays the scaled FGDI values for all amputees in the sample, categorized by their K-Level. A Wilcoxon rank sum exact test confirms that the median scaled FGDI values are significantly higher for individuals classified as K2 compared to those classified as K3, resulting in p-values of 0.03 for the amputated side and 0.009 for the non-amputated side.

## 5. A comparison of the FGDI with existing approaches

To facilitate comparison with the GDI approach, we recalculated the FGDI at 51 equally-spaced time points within a single gait cycle, corresponding to increments of 2%.

### 5.1. The combined leg approach

The FGDI, GPS, and OA indices are utilized to assess gait abnormalities in PD patients, considering both legs collectively. Kendall’s rank correlation coefficient is employed to quantify the association between any pair of indices. The correlation coefficients for the indices are as follows: scaled FGDI vs GPS is 0.54, scaled FGDI vs OA is 0.42, and GPS vs OA is 0.34. The scaled FGDI demonstrates a moderate correlation with both GPS and OA, indicating that while these indices similarly measure gait pathology, they differ in their specific rankings of gait abnormalities. The weaker correlation between GPS and OA suggests a more pronounced difference in their assessment of gait pathology.

**Figure 5.**
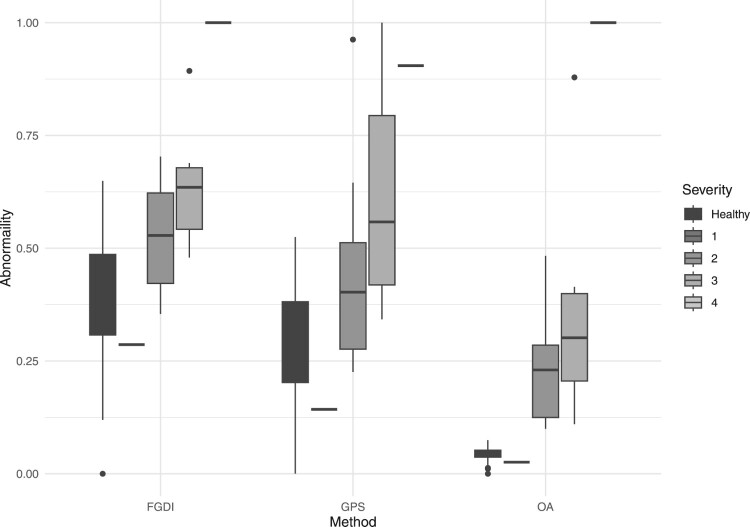
Gait abnormality on both legs relative to the Hoehn and Yahr scale for the FGDI, GPS and OA methods respectively.

Figure 5 presents boxplots that depict the abnormality in both legs for all subjects relative to the Hoehn and Yahr scale, utilizing the FGDI, GPS, and OA methods. To facilitate direct comparisons among these indices, each severity measure is rescaled to have values ranging from 0 to 1. On this scale, a score of 0 indicates the least abnormal gait, and a score of 1 represents the most abnormal gait.

Consistent with expectations, all methods show increased gait abnormality at higher levels of the Hoehn and Yahr scale. A one-way Kruskal-Wallis rank sum test was implemented to determine if the median values for different Hoehn and Yahr scale levels significantly differ for each method, with p-values of 0.08, 0.09 and 0.09 for FGDI, GPS, and OA respectively. It is anticipated that the subject with the most severe gait abnormality corresponds to the highest Hoehn and Yahr scale level, i.e. level 4. Both the FGDI and OA methods correctly identify the subject at the highest Hoehn and Yahr level as having the most abnormal gait. However, the GPS method assigns a subject with a Hoehn and Yahr scale of 3 as having the most abnormal gait and gives a disproportionately high score to a subject with a Hoehn and Yahr scale of 2.

**Figure 6.**
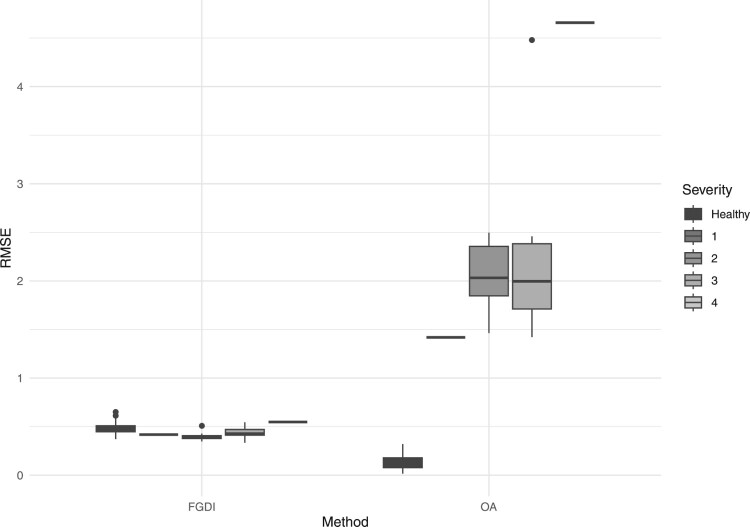
The approximation error relative to the Hoehn and Yahr scale for all subjects for the FGDI and OA methods.

The OA and FGDI methodologies both utilize an independent gait feature space to calculate their respective measures of gait pathology. To assess the suitability of this space, the accuracy of gait movement approximations, achieved through basis function expansion within this space, can be evaluated. The OA and FGDI must accurately reflect the severity of walking conditions; hence, the corresponding gait movement approximations should capture essential aspects of gait, including significant timing differences, substantial level shifts, and pattern alterations. If the gait movement approximations fail to encompass these critical features, the corresponding measures of gait pathology will also lack this crucial information. The accuracy of each gait movement approximation is assessed by calculating the average root-mean-squared error (RMSE) between the kinematic data and the approximated curve ${\hat{m}}_{i,u}(t)$ for each subject. See Appendix 3 for details on the calculation of this metric. Figure 6 illustrates the boxplots of the average of the RMSE across each kinematic variable relative to the Hoehn and Yahr scale for all subjects.

Figure 6 illustrates that the FGDI method produces a low approximation error across all stages of the Hoehn and Yahr scale. In contrast, the OA method displays significant differences in the approximation error of healthy individuals and those with PD. In addition, the average RMSE for all joints/planes in PD subjects is 0.46 for FGDI and 0.83 for OA. Consequently, the FGDI method enhances the accuracy of gait movement approximations in PD patients compared to the OA method.

### 5.2. The individual leg approach

To individually assess the severity of gait abnormalities in both the left and right legs, methodologies such as the scaled FGDI, GDI, GPS, and OA can be employed. These methods are utilized to evaluate gait abnormalities in individuals with lower-limb amputations. Kendall’s rank correlation coefficients for these indices are provided in Table 1.

**Table 1.** Kendall’s rank correlation for each pair of rankings for the left and right leg separately.

	sFGDI vs GDI	sFGDI vs GPS	sFGDI vs OA	GDI vs GPS	GDI vs OA	GPS vs OA
Left	−0.93	0.95	0.55	−0.93	−0.54	0.57
Right	−0.94	0.95	0.54	−0.93	−0.51	0.55

**Figure A2.**
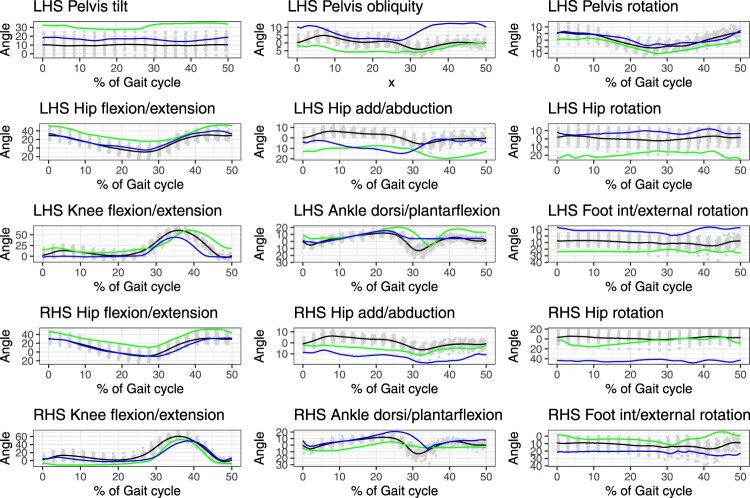
The gait patterns of 42 healthy subjects (represented in grey) and their mean (depicted in black). Additionally, it displays the gait pattern of the subject identified as having the most abnormal gait by scaled FGDI, GDI, and GPS (highlighted in green), as well as the gait pattern of the subject identified as having the most abnormal gait by OA (highlighted in blue).

The sFGDI demonstrates a strong correlation with both the GDI and GPS, while the OA method reveals a more pronounced disparity in the ranking of gait abnormalities. The scaled FGDI, GDI, and GPS consistently identify the same individual as having the most severe gait abnormalities on both the left and right sides, corresponding with their K2 classification. Conversely, the OA approach inaccurately identifies an individual with a K3 classification as exhibiting the most abnormal movement on the left side. Figure A2 in Appendix 5 illustrates the gait patterns of 42 healthy subjects (represented in grey) alongside their mean (depicted in black). Additionally, it highlights the gait pattern of the subject identified as having the most abnormal gait by the scaled FGDI, GDI, and GPS (in green), as well as the gait pattern of the subject identified by the OA method (in blue). A detailed examination of the gait data on the left-hand side clearly shows that the green curves exhibit less similarity to the black curves than the blue curves.

A Wilcoxon rank sum test indicates that for the Amputated side, the median values of the GDI, GPS, and OA do not show significant variation between K-Levels (GDI p-value: 0.09, GPS p-value: 0.06; OA p-value: 0.17), but they do on the non-Amputated side (GDI p-value: 0.03; both GPS and OA p-value is 0.01). For the FGDI the corresponding p-values are 0.02 for the amputated side and 0.01 for the non-amputated side.

## 6. The FGDI interactive web app

A free R Shiny application has been developed to serve as a graphical user interface (GUI) and is accessible at the following URL: https://michelle-carey-ucd.shinyapps.io/FGDI_ShinyApp/. This GUI enables users to upload their gait data and computes the scaled FGDI, presenting several outputs:

- The scaled FGDI for the left, right, and both legs for a user-selected subject.
- A comparison of the average typically developing gait versus the gait of a user-selected subject, akin to that shown in Figure 1.
- A comparison between observed and approximated gait for a user-selected subject, allowing the user to visually assess the quality of the gait approximation.
- The MAP summarizing the abnormality of each of the 15 kinematic variables for a user-selected subject, similar to Figure 2.
- A comparison of the MAP between two subjects for a pair of user-selected subjects.

## 7. Conclusion

The GDI and GPS are well-established indices for measuring gait abnormalities. However, as discussed in [16], the non-independent nature of kinematic data can lead to biased assessments of gait pathology using these indices. While the OA approach was developed to address this issue, it fails to account for the inherent dependence structure. The time-ordered nature of kinematic data throughout the gait cycle is crucial in gait analysis and must be preserved. It is the interdependence across joints and planes that contributes to multicollinearity, and thus biases measures of gait pathology.

We introduce the FGDI, which utilizes MFPCA to establish an independent gait feature space. The FGDI calculates the distance between a subject’s projected kinematics and the control group’s average projection within this space. This index captures the intrinsic smoothness of gait movements across each joint and plane, and it accounts for potential covariation among them. FGDI is correlated with overall gait function, providing a reliable measure of gait abnormality. Furthermore, it is readily accessible through an interactive web application and R package.

The key distinctions between the GDI and the FGDI are as follows: (i) The GDI derives its independent feature space from a PCA of a large gait database, whereas the FGDI employs MFPCA on sampled data to construct its feature space. (ii) The GDI’s PCA identifies primary modes of variation by simultaneously considering time and kinematic variables. In contrast, the FGDI treats the movement of each kinematic variable as distinct curves. By applying MFPCA to these curves, the FGDI specifically aims to capture key variations in movement patterns throughout the gait cycle while accounting for any co-variation between the kinematic variables.

The FGDI, GPS, and OA indices are used to evaluate gait abnormalities in PD patients, considering both legs collectively. The scaled FGDI aligns well with the Hoehn and Yahr scale, it also effectively distinguishes between individuals classified as freezers or non-freezers and shows an increasing trend relative to Parts II and III of the MDS-UPDRS. In contrast, the GPS method does not consistently align with the Hoehn and Yahr scale. For instance, it incorrectly identifies a subject with a Hoehn and Yahr scale of 3 as having the most abnormal gait and assigns an unusually high score to a subject with a Hoehn and Yahr scale of 2.

For the scaled FGDI approach, the average RMSE across all joints shows relative consistency with respect to the the Hoehn and Yahr scale, with values of 0.58 for the healthy group, 0.41 for scale 1 individuals, 0.39 for scale 2, 0.43 for scale 3, and 0.54 for scale 4. However, the OA approach demonstrates a significant increase in gait approximation error with advancing Hoehn and Yahr scales. The average RMSE values are 0.13 for the healthy group, escalating to 1.42 for scale 1, 2.06 for scale 2, 2.30 for scale 3, and reaching 4.66 for scale 4 individuals. The OA method, which relies on a gait feature space constructed solely from individuals without gait abnormalities, faces challenges in accurately capturing the complex gait patterns of individuals with higher Hoehn and Yahr scales. This limitation in the gait feature space means that the principal component scores derived from the OA method lack critical information about the movement of individuals with higher Hoehn and Yahr scales, resulting in a biased assessment of gait pathology.

To evaluate the severity of gait abnormalities in both the left and right legs individually, methodologies such as the scaled FGDI, GDI, GPS, and OA can be employed. Given that PD typically manifests with unilateral motor symptoms, we focus on assessing gait abnormalities in individuals with lower-limb amputations for each leg separately. The scaled FGDI values are notably higher for K2 compared to K3, both on the amputated and non-amputated sides. The scaled FGDI, GDI, and GPS consistently identify individuals with the most severe gait abnormalities, aligning with their K2 classification on both the left and right sides. Conversely, the OA approach inaccurately identifies an individual with a K3 classification as having the most abnormal movement on the left side.

Providing the scaled FGDI alongside standard measures of gait abnormalities (e.g. the Hoehn and Yahr scale, MDS-UPDRS Parts II and III, K-Level) offers multiple advantages. The scaled FGDI provides a quantitative measurement of gait quality, enabling more precise and detailed assessments of gait abnormalities compared to the ordinal scales typically employed by standard measures. Additionally, the scaled FGDI is sensitive enough to detect subtle changes in gait quality over time, making it useful for monitoring progress and evaluating the effectiveness of interventions or treatments. The FGDI approach yields specific information about affected joints, facilitating the development of tailored treatment plans. Moreover, the scaled FGDI is more objective as it relies on recorded kinematic data of the patient’s movement, in contrast to clinical observations or patient-reported severity, which introduce subjectivity into the assessment process.

Future research will involve incorporating covariates such as the individual’s sex and age into the MFPCA approach. This inclusion aims to determine if there are discernible differences in the scaled FGDI relative to these variables and to develop a metric that incorporates these variables when quantifying the severity of an individual’s gait pattern.

## Data Availability

The datasets mentioned in this study are publicly available. The Parkinson’s Disease dataset is accessible via [27], while the healthy dataset can be found in [6]. Additionally, the lower-limb amputation dataset is accessible through [14]. The data and R code for reproducing the results in this article are available at fdaatucd.com or in the fgdiR R Package.

## Note

1 Further details on the dataset are provided in Section 3

## Appendix 1. Existing approaches for quantifying gait pathology

Three of the most popular indices for quantifying gait pathology are: the GDI, the GPS, and the OA measure. This section briefly reviews each of these approaches.

### A.1. The gait deviation index

One barefoot stride was selected from each side of the 3, 351 subjects observed at the Gillette Children’s Specialty Healthcare Center for Gait and Motion Analysis between February 1994 and April 2007, totaling 6, 702 sides. These data are organized into a vector g comprising of 459 observations that pertain to the 9 kinematic variables evaluated across 51 equally spaced points in the gait cycle. For example, $g_{1}=[m_{1,1}^{*},m_{1,2}^{*},\ldots ,m_{1,9}^{*}{]}^{T}$ is the data for subject 1 pertaining to the nine kinematic variables on the left-hand side and $g_{2}=[m_{1,10}^{*},m_{1,11}^{*},\ldots ,m_{1,18}^{*}{]}^{T}$ contains the data for the same subject on the right-hand side, with $m_{i,j}^{*}$ representing a length 51 vector of observations for the *i*th individual in the database and *j*th kinematic variable. We introduce the notation $m^{*}$ to distinguish between data from the database and data from the sample, denoted previously as *m*. Note that there are two vectors for each subject one for the left-hand side and one for the right-hand side. These vectors are combined to form a 459×6702 gait matrix. A singular value decomposition was performed on this gait matrix to obtain a 459×15 matrix containing a reduced set of independent gait features (i.e. the left singular vectors) $\hat{f}=[{\hat{f}}_{1},{\hat{f}}_{2},\ldots ,{\hat{f}}_{15}]$. These length 15 singular vectors form an orthonormal basis that accounts for 98% of the total variation in the Gillette gait data. The original data are not publicly available but the gait features are in the supplementary material of [26].

We sample the observed data at 51 time points for nine kinematic variables on one selected side (either left or right) and combine them into a vector $q_{i}$ of length 459. For illustration, we consider the left side, where $q_{i}=[m_{i,1},m_{i,2},\ldots ,m_{i,9}{]}^{T}$, with $m_{i,j}$ representing a length 51 vector of observations for the *i*th individual in the sample and *j*th kinematic variable. The feature components $c_{i,k}$, which projects the gait vector $q_{i}$ onto the *k*th feature direction, are given by $c_{i,k}=q_{i}^{T}\hat{f_{k}},$ where k=1,…,15. Let $c_{i}=[c_{i,1},\ldots ,c_{i,15}]$.

To calculate the GDI, evaluate the log of the Euclidean distance between the gait features for the *i*th subject, $c_{i}$, relative to the average gait features derived from all typically developing subjects, ${\bar{c}}_{\mathrm{TD}}.$ That is, ${\mathrm{GDI}}_{i}=\log(\|c_{i}-{\bar{c}}_{\mathrm{TD}}\|).$ The GDI offers separate measures of severity for the left and right legs.

Compute the sample mean and standard deviation of the GDI for the typically developing subjects denoted by $\mu_{{\mathrm{GDI}}_{\mathrm{TD}}}$ and $\sigma_{{\mathrm{GDI}}_{\mathrm{TD}}}$ respectively. To improve interpretation the following scaled GDI measure is often reported, ${\mathrm{sGDI}}_{i}=100-10\times \frac{{\mathrm{GDI}}_{i}-\mu_{{\mathrm{GDI}}_{\mathrm{TD}}}}{\sigma_{{\mathrm{GDI}}_{\mathrm{TD}}}}.$ Every 10 points, the scaled GDI falls below 100 corresponds to one standard deviation away from the typically developing mean.

### A.2. The gait profile score

The Gait Variable Score (GVS), is the root-mean-square difference between the gait vector $m_{i,j}$ and the average of the gait vectors for kinematic variable *j*, for all typically developing subjects. The GPS is obtained by evaluating the root-mean-square of the GVS across all kinematic variables for a particular side. To obtain an overall GPS, we calculate the root-mean-square average of the GPS values from both the left and right sides. Given that the pelvis is common to both sides, we include pelvic kinematics from only one side. This results in a total of 15 included kinematic variables: nine on the left side and six on the right side.

### A.3. The overall abnormality

The OA metric can be calculated using two different approaches. The first method involves combining the observations of nine kinematic variables for the left side and six for the right side, resulting in a measure of abnormality that considers both legs collectively. Alternatively, the OA metric can be calculated separately for each leg by using the observations of the nine kinematic variables specific to that leg. This approach provides an individual severity measure for each leg.

The observations of the kinematic variables for subject *i* are combined into a length M=D×T vector $q_{i}=[m_{i,1},m_{i,2},\ldots ,m_{i,D}{]}^{T}$. The length of this vector depends on the number of joints or angles included, either *D* = 15 or *D* = 9 as described in Section 2.2 with *T* denoting the number of observed points in the gait cycle typically *T* = 101. The vectors $q_{i}$ for the typically developing subjects are combined to form a $N_{\mathrm{TD}}\times M$ gait matrix, $G=[q_{1}\ldots q_{N_{\mathrm{TD}}}{]}^{T},$ where $N_{\mathrm{TD}}$ is the number of typically developing subjects in the sample. Let $\tilde{G}$ be the matrix G shifted to be zero-centered and scaled to have unit variance. A PCA is performed on $\tilde{G}$ to obtain a M×K matrix containing a reduced set of independent gait features (eigen-vectors), $\hat{r}=[{\hat{r}}_{1},{\hat{r}}_{2},\ldots ,{\hat{r}}_{K}],$ and corresponding eigenvalues $\gamma_{1},\ldots ,\gamma_{K}$. The number of principal components *K* is chosen in accordance with the number of eigenvalues greater than or equal to one as recommended in [10,15].

Calculate the mean, $\mu_{\tilde{G}},$ and the standard deviation, $\sigma_{\tilde{G}},$ of $\tilde{G}$. Re-scale the *i*th subjects kinematic data ${\tilde{q}}_{i}=\frac{q_{i}-\mu_{\tilde{G}}}{\sigma_{\tilde{G}}}.$ One can then compute the feature components $s_{i,k}$ which project any scaled gait vector ${\tilde{q}}_{i}$ onto the *k*th feature direction by, $s_{i,k}={\tilde{q}}_{i}^{T}\hat{r_{k}},$ where k=1,…,K. The *i*th subjects’ abnormality is measured by

$$A_{i}=\frac{1}{K}\sum_{k=1}^{K}\left|\frac{s_{i,k}-{\bar{s}}_{\mathrm{TD}}}{\sigma_{\mathrm{TD}}}\right|,$$

where ${\bar{s}}_{\mathrm{TD}},$ is the average gait features derived from all subjects without gait abnormalities and $\sigma_{\mathrm{TD}}$ is the standard deviation of the gait features derived from all subjects without gait abnormalities.

## Appendix 2. A note on the distribution of FGDI

The functional principal components are assumed to be normally distributed if the gait data is generated from a mean-zero Gaussian process; for further details, see [11]. Let $\left({\hat{\varphi }}_{i,p}-{\bar{\hat{\varphi }}}_{p}^{\left(H\right)})∼N_{p}(0,\Sigma \right)$, where Σ is a positive definite covariance matrix. By the spectral decomposition of Σ, we can write $\Sigma =Q\Lambda Q^{⊤},$ where $\Lambda =\mathrm{diag}(\lambda_{1},\ldots ,\lambda_{k})$ are the eigenvalues of Σ, and Q is an orthogonal matrix of eigenvectors. Then $({\hat{\varphi }}_{i,p}-{\bar{\hat{\varphi }}}_{p}^{\left(H\right)})=\Sigma^{1/2}Z$, where $Z∼N_{p}(0,I)$, and we obtain $({\hat{\varphi }}_{i,p}-{\bar{\hat{\varphi }}}_{p}^{\left(H\right)}{)}^{2}=\sum_{j=1}^{k}\lambda_{j}Z_{j}^{2},$ where $Z_{j}∼N(0,1)$ are i.i.d. standard normal variables. Thus, $d_{i,p}∼\sum_{j=1}^{k}\lambda_{j}\chi_{1,j}^{2}$, a generalized chi-squared distribution, i.e. a linear combination of independent chi-squared random variables with 1 degree of freedom. The Euclidean norm is given by $\sqrt{d_{i,p}}$. This has the distribution of the square root of a generalized chi-squared variable and does not have a closed-form expression in general. This distribution is non-negative, continuous and has a heavy tail if the largest eigenvalue dominates. Therefore, a logarithmic transformation is applied to render the distribution more symmetric so that standard nonparametric tests can be used to assess differences between groups.

## Appendix 3. Approximation error

Recall that the dataset is represented by $m_{i,u,l}$, where *u* ranges from 1 to *U*, covering various kinematic variables. The index *i* represents individual subjects, numbering from 1 to *N*, and *l* indicates the observed points throughout the gait cycle, from 1 to *T*.

The estimated gait function for kinematic variable *u* for subject *i*, ${\hat{m}}_{i,u}(t),$ can be calculated by the univariate functional Karhunen-Loéve approximation of the data,

(A1)
$${\hat{m}}_{i,u}(t)\approx {\hat{\mu }}_{u}(t)+\sum_{w=1}^{W}{\hat{\xi }}_{i,w}^{u}{\hat{\phi }}_{w}^{u}(t),$$

where ${\hat{\mu }}_{u}(t)$ is an estimate for the mean function for the kinematic variable *u* across all subjects, ${\hat{\xi }}_{i,w}^{u}$ is the *w*th univariate principal component score for the *i*th subject and ${\hat{\phi }}_{w}^{u}(t)$ is the *w*th univariate principal component function for the *u*th kinematic variable.

The root mean square error (RMSE) between the kinematic data $m_{i,u,l}$ and the approximated curve ${\hat{m}}_{i,u}(t)$ for each subject and kinematic variable is calculated as follows:

(A2)
$${\mathrm{RMSE}}_{i,u}=\sqrt{\sum_{l=1}^{T}\frac{(m_{i,u,l}-{\hat{m}}_{i,u}(t_{l}){)}^{2}}{T}}$$

The average value across all kinematic variables is then ${\mathrm{RMSE}}_{i}=\sum_{i=1}^{U}\frac{{\mathrm{RMSE}}_{i,u}}{U}.$

## Appendix 4. Stability of FGDI relative to the number of principal components

We examine the stability of the FGDI in relation to the number of principal components using both the joint plane-specific and combined approaches. Let δ±j represent 100 times the difference between the FGDI calculated with the optimal number of principal components and the FGDI obtained when the number of principal components is adjusted by ±j. The optimal number of principal components and the corresponding values for $\delta_{-2}$, $\delta_{-1}$, $\delta_{+1}$, and $\delta_{+2}$ for each kinematic variable are detailed in Table A1.

**Table A1.** The number of principal components selected for each kinematic variable.

Kinematic Variable	No. PCs	$\delta_{-2}$	$\delta_{-1}$	$\delta_{+1}$	$\delta_{+2}$
LHS ankle dorsiflexion/plantarfexion	10	−0.26	0.22	−0.16	−0.16
LHS foot int/external rotation	8	−3.06	−0.07	−0.03	−0.01
LHS hip abduction/adduction	7	−0.50	0.04	0.00	−0.04
LHS hip flexion/extension	4	−0.50	0.04	0.00	−0.04
LHS hip rotation	5	0.17	0.24	−0.07	−0.05
LHS knee flexion/extension	7	−0.60	−0.36	−0.04	−0.07
LHS pelvic obliquity	7	−0.69	−0.15	−0.02	0.08
LHS pelvic rotation	7	−0.32	−0.13	0.34	0.42
LHS pelvic tilt	3	0.30	0.15	−0.25	−0.32
RHS ankle dorsiflexion/plantarflexion	10	−2.21	−1.87	0.38	0.56
RHS foot int/external rotation	8	0.14	0.09	−0.04	−0.01
RHS hip abduction/adduction	7	−0.38	−0.19	−0.05	−0.03
RHS hip flexion/extension	4	−0.82	−0.23	−0.03	0.07
RHS hip rotation	5	−0.41	−0.09	0.13	0.17
RHS knee flexion/extension	7	0.36	0.23	−0.20	−0.24

Table A1 demonstrates that the joint plane-specific FGDI remains highly stable across different numbers of principal components, with minimal variations in values. Figure A1 illustrates 100 times the difference between the combined FGDI calculated with the optimal number of principal components and when the number is adjusted by ±5. As shown, there are minimal differences in combined FGDI values with changes in the number of principal components.

## Appendix 5. Comparison of methods

Figure A2 illustrates the gait patterns of 42 healthy subjects (represented in grey) and their mean (depicted in black). Additionally, it displays the gait pattern of the subject identified as having the most abnormal gait by sFGDI, GDI, and GPS (highlighted in green), as well as the gait pattern of the subject identified as having the most abnormal gait by OA (highlighted in blue).

A detailed examination of the gait data on the left-hand side clearly shows that the green curves exhibit less similarity to the black curves than the blue curves.
